# Anti-inflammatory effects of *Morus alba* Linne bark on the activation of toll-like receptors and imiquimod-induced ear edema in mice

**DOI:** 10.1186/s12906-021-03291-5

**Published:** 2021-04-09

**Authors:** Lin Umeyama, Besse Hardianti, Shiori Kasahara, Dya Fita Dibwe, Suresh Awale, Satoru Yokoyama, Yoshihiro Hayakawa

**Affiliations:** 1 Institute of Natural Medicine, University of Toyama, 2630 Sugitani, Toyama, 930-0194 Japan; 2 Sekolah Tinggi Ilmu Farmasi Makassar, Perintis Kemerdekaan Street Km 13.7, Makassar, 90242 Indonesia; 3 Department of Cancer Cell Biology, Graduate School of Medicine and Pharmaceutical Science, University of Toyama, 2630 Sugitani, Toyama, 930-0194 Japan

**Keywords:** Inflammation, Toll-like receptor, Psoriasis, Innate immunity, *Morus alba* L. bark

## Abstract

**Background:**

*Morus alba* L. bark has been widely used in traditional medicine for treating several inflammatory diseases, such as hypertension, diabetes mellitus and coughing; however, the molecular mechanisms underlying its anti-inflammatory effects are not well understood.

**Methods:**

We examined the effects of an extract of *Morus alba* L. bark (MabE) on Toll-like receptor (TLR) ligand-induced activation of RAW264.7 macrophages using a luciferase reporter assay and immunoassays. For the in vivo experiment, we used an imiquimod-induced ear edema model to examine the anti-inflammatory effects of MabE.

**Results:**

MabE inhibited the TLR ligand-induced activation of NF-κB in RAW264.7 cells without affecting their viability. Consistent with the inhibition of NF-κB activation, MabE also inhibited the production of IL-6 and IL-1β from TLR ligand-treated RAW264.7 cells. In vivo MabE treatment inhibited the ear swelling of IMQ-treated mice, in addition to the mRNA expression of IL-17A, IL-1β and COX-2. The increases in splenic γδT cells in IMQ-treated mice and the production of IL-17A from splenocytes were significantly inhibited by MabE treatment.

**Conclusion:**

Our study suggests that the anti-inflammatory effects of MabE on the activation of the macrophage cell line RAW246.7 by TLRs and IMQ-induced ear edema are through the inhibition of NF-κB activation and IL-17A-producing γδT cells, respectively.

**Supplementary Information:**

The online version contains supplementary material available at 10.1186/s12906-021-03291-5.

## Backgrounds

Inflammation is one of the host defense mechanisms against pathogenic stimulation such as physical irritation or infections. A series of biological processes is involved in inflammation including the destruction and removal of pathogenic substances and subsequent repair of damaged tissues [1]. In recent years, the incidence of aging-associated diseases, such as cancer, metabolic diseases, neurodegenerative diseases and autoimmune diseases has increased, and the caues and aggravation of these aging-associated diseases are related to chronic inflammation [2–4]. Therefore, sufficient control of abnormal inflammation is considered to be important to prevent and treat aging-associated diseases.

Nuclear factor-kappa B (NF-κB) is an important transcription factor that plays a central role in inflammation by controlling the expression of inflammation-associated molecules such as pro-inflammatory cytokines, adhesion molecules and chemokines [5, 6]. Furthermore, NF-κB is essential not only for initiating inflammation, but also for maintaining it; therefore, the regulation of NF-κB activity is required for maintaining tissue homeostasis [5, 7]. The activation of NF-κB is induced by the pathogen recognition mechanism via pattern recognition receptors (PRRs) [8]. Among the variety of PRRs, Toll-like receptors (TLRs) are one of the primary PPRs and have been extensively investigated [9, 10]. TLRs recognize not only microbe-derived components, termed pathogen-associated molecular patterns (PAMPs), such as lipopolysaccharide (LPS), peptidoglycan and double stranded RNA (dsRNA), but also self-derived components, referred to as damage-associated molecular patterns (DAMPs), released from damaged cells [11]. To date, 10 types of TLRs (TLR1–10) have been found in humans and 12 types (TLR1–9, 11–13) have been found in mice. Although the cell types and location of TLR expression may vary depending on the receptor type [12, 13], there are many TLRs expressed on immune cells, such as macrophages, dendritic cells and neutrophils, that play important roles in the initiation of innate immunity [11].

Among the numerous cytokines cytokines and immune cells, the inflammatory cytokine IL-17A and its source Th17 and/or γδT17 cells are known to be required for the development inflammatory diseases [14, 15]. IL-17A induces STAT3-dependent proliferative and anti-apoptotic gene expression promoting epidermal cell proliferation and hyperplasia [16]. In addition, IL-17 is involved in the induction of chemokine expression to attract inflammatory cells [17, 18]; therefore, IL-17A and its source are rconsidered pharmacological targets for treating inflammatory diseases.

*Morus alba* L. bark is a dried herbal bark of the mulberry family, and is a traditional natural medicine called “Sohakuhi” in Japan which is known to have diureitc, blood pressure- and blood glucose-reducing, antipyretic and antitussive effects. Several Kampo medicines, such as Seihaito or Gokoto, contain Sohakuhi as an active constituent, and these Kampo medicines have been used for treating severe cough or bronchial asthma [19, 20]. We recently identified a fraction of the ethyl acetate extract of *Morus alba* L. bark (MabE) inhibit NF-κB activity in breast cancer cells and keratinocytes treated with TRAIL [21]. In this study, we examined the anti-inflammatory effects of MabE on the activation of TLRs in the RAW264.7 macrophage cell line and imiquimod-induced ear edema model mice.

## Methods

### Experimental materials

The extract of *Morus alba* L. bark (Sohakuhi) was prepared as described previously [21]. Briefly, *Morus alba* L. bark (1 kg, Sohakuhi/CD14071, Tochimoto Tenkaido Co., Ltd., Osaka, Japan) was refluxed in MeOH (5 L, 90 min, × 2) to obtain the MeOH extract, and subsequently suspended in water (500 mL) and defatted by Hexane (500 mL, × 3) to separate the aqueous layer. The aqueous layer was further extracted with EtOAc (500 mL, × 3) to obtain EtOAc layer. This EtOAc layer was used as an extract of *Morus alba* L. bark (MabE). Moracin O and P were isolated from MabE using a CHCl_3_-MeOH gradient RP-MPLC fractionation system with mass spectrometry and ^1^H-NMR analysis. The extract and compounds were dissolved in dimethyl sulfoxide (DMSO) for in vitro study. For in vivo study, the extract was further diluted in 50% EtOH.

### Cells

RAW264.7 cells, a mouse monocytic leukemia-derived macrophage cell line (ATCC, VA, USA), and RAW264.7-NFκB-Luc2 cells were established accordingly [22], and cultured in DMEM (Nissui Seiyaku, Tokyo, Japan) containing 10% fetal bovine serum (FBS, Nichirei Biosciences, Tokyo, Japan), 1 mM L-glutamine (Life Technologies, Gaithersburg, MD, USA), 0.2% NaHCO_3_ and antibiotics (100 units/mL of penicillin and 100 mg/mL of streptomycin) in a humidified atmosphere of 95% air and 5% CO_2_ at 37 °C.

### Cell viability assay

RAW264.7-NFκB-Luc2 cells were seeded in a 96-well plate at 2 × 10^4^ cells / well and cultured at 37 °C overnight, then MabE (0, 0.5, 1, 5, 10, or 25 μg/mL) or Moracin O or P (0, 0.3, 3, 10, 100, or 1000 nM) was added. After incubation for 1 h, the cells were stimulated with IMQ (10 μg/mL), polyI:C (10 μg/mL), and LPS (10 ng/mL) and cultured at 37 °C for 24 h. Thereafter, WST-1 reagent (Dojindo, Japan) was added and the absorbance was measured at a wavelength of 450 nm. Cell viability was compared with that of an unstimulated control group.

### Luciferase reporter assay

RAW264.7-NFκB-Luc2 cells were seeded in a 96-well plate at 5 × 10^4^ cells/well and cultured at 37 °C overnight, then MabE (0, 0.5, 1, 5, 10, or 25 μg/mL) or Moracin O, P (0, 0.3, 3, 10, 100, or 1000 nM) were added. After a 1-h incubation, the cells were stimulated with IMQ (10 μg/mL), polyI:C (10 μg/mL), LPS (10 ng/mL), and 20 μL/well of luciferin was immediately added. Luminescent activity after 6 h was measured using IVIS LUMINA II (Caliper Life Sciences).

### Real-time PCR

Total RNA of RAW264.7 cells was prepared using the RNeasy Plus Mini kit (Qiagen, Hilden, Germany). Total mRNA was extracted from whole biopsy of the ear after sacrificing the mice using TRIZOL reagent (Invitrogen) according to the manufacturer’s instructions. Expression levels of IL-6, IL-1β, IL-17A and COX-2 mRNA were measured by real-time PCR using the ABI Prism 7300 sequence detection system (Life Technologies Corporation, Garlsbad, CA, USA) and normalized to GAPDH mRNA. The primers used were: 5′- CTGGAGCCCACCAAGAACGA-3′ (forward) and 5′- GCCTCCGACTTGTGAAGTGGT-3′(reverse) for IL-6 mRNA, 5′- TCCAGGATGAGGACATGAGCAC-3′ (forward) and 5′- GAACGTCACACACCAGCAGGTTA-3′ (reverse) for IL-1β mRNA, 5′- CAC CTC ACA CGA GGC ACA AG-3′ (forward), and 5′-GCA GCA ACA GCA TCA GAG ACA-3′ (reverse) for IL-17A, 5′-GTG TGC GAC ATA CTC AAG CAG GA-3′ (forward), and 5′-TGA AGT GGT AAC CGC TCA GGT G-3′ (reverse) for COX-2, 5′- AAATGGTGAAGGTCGGTGTG-3′ (forward) and 5′- TGAAGGGGTCGTTGATGG-3′(reverse) for GAPDH mRNA.

### Measurement of cytokines

RAW264.7 cells were seeded in 24-well plates (10^5^ cells per well) and incubated overnight. The cells were treated with MabE (50 μg/mL) for 1 h before stimulation with TLR ligands (IMQ: 20 μg/mL, polyI:C: 20 μg/mL, and LPS: 100 ng/mL). After 24 h, supernatants were collected and stored at − 80 °C until use. IL-6 and IL-1β secretion was quantified using the specific ELISA kit (BioLegend) according to the manufacturer’s instructions.

### Western blotting

Cell lysates were collected in lysis buffer (25 mM HEPES pH 7.7, 0.3 M NaCl, 1.5 mM MgCl_2_, 0.2 mM EDTA, 0.1% TritonX-100, 20 mM β-glycero-phosphate, 1 mM sodium orthovanadate, 1 mM phenyl-methylsulfonyl fluoride, 1 mM dithiothreitol, 10 mg/mL of aprotinin, and 10 mg/mL of leupeptin). Equal amounts of protein were resolved by electrophoresis on 10% acrylamide gel and transferred to polyvinylidene difluoride (PVDF) membranes. The primary antibodies used were p65, p-p65 (S536), p-ERK (T202/Y204), p-p38 (T180/Y182), (Cell Signaling Technology, Beverly, MA, USA), and ERK1/2, p38α and β-actin (Santa Cruz Biotechmology, Santa Cruz, CA, USA).

### Imiquimod (IMQ)-induced psoriasis model

Female Balb/c mice (6–7 week old, approximately 18 ~ 19 g weight) were purchased from Japan SLC (Hamamatsu, Japan). All experiments were approved and performed according to the guidelines of the Care and Use of Laboratory Animals of the University of Toyama. For anesthetic methods, the inhalation dose of 1.5 ~ 2.0% of isoflurane (Pfizer, NY, USA) was used in this study. To terminate the experiments, mice were sacrificed by cervical dislocation under the anesthesia.

An experimental psoriasis model was established by following the methods of van der Fits et al. [23] with modification. Balb/c mice received a daily topical dose of 20 μg of commercially available IMQ cream (5%) (Aldara; Mochida Pharmaceuticals) on the right ear for five consecutive days, equivalent to a daily dose of 1 μg of the active compound. Ear thickness was measured by a thickness gauge 24 h after each IMQ challange. A group of mice was topically treated with MabE (200 μg in 20 μL of 50% EtOH) on the right ear 30 min before IMQ challenge.

### Flow cytometry

Spleens from Balb/c mice were minced in RPMI1640 medium and passed through 80-μm mesh after mechanical disruption, and cells were treated with RBC lysis buffer to deplete red blood cells. For flow cytometry analysis, cells were first preincubated with anti-CD16/32 (2.4G2) to prevent nonspecific binding of antibodies to FcγR. Then, the cells were incubated with a saturating amount of fluorophore-conjugated monoclonal antibody (mAb). Antibodies against CD3ε (2C11), CD4 (RM4–5), NKp46 (29A1.4) and γδTCR (GL3) were purchased from BD Bioscience or Biolegend. Flow cytometry analysis was performed with a FACS Canto (BD Bioscience) and the data were analyzed using FlowJo software (Tree Star).

### Measurement of IL-17A production

Spleens from Balb/c mice were minced in RPMI1640 medium and passed through 80-μm mesh after mechanical disruption, and cells were treated with RBC lysis buffer to deplete red blood cells. The splenocytes were cultured at a density of 2 × 10^6^ cells/well in 24-well plates with MabE or culture medium. After 1 h, cells were stimulated with PMA (10 ng/mL) / Ionomycin (500 ng/mL) for 24 h. The cell-free culture supernatants were collected and stored at − 80 °C until use. IL-17A secretion was quantified using the specific ELISA kit (BioLegend) according to the manufacturer’s instructions.

### Statistical analysis

All data are expressed as the mean ± SEM of at least two independent experiments unless otherwise stated. Significance was analyzed using two-way ANOVA and Bonferroni post tests using SPSS- 25. *P* < 0.05 was considered significant.

## Results

### MabE inhibits the TLR ligand-induced activation of inflammatory signals and cytokine production in RAW264.7 cells

**Fig. 1:**
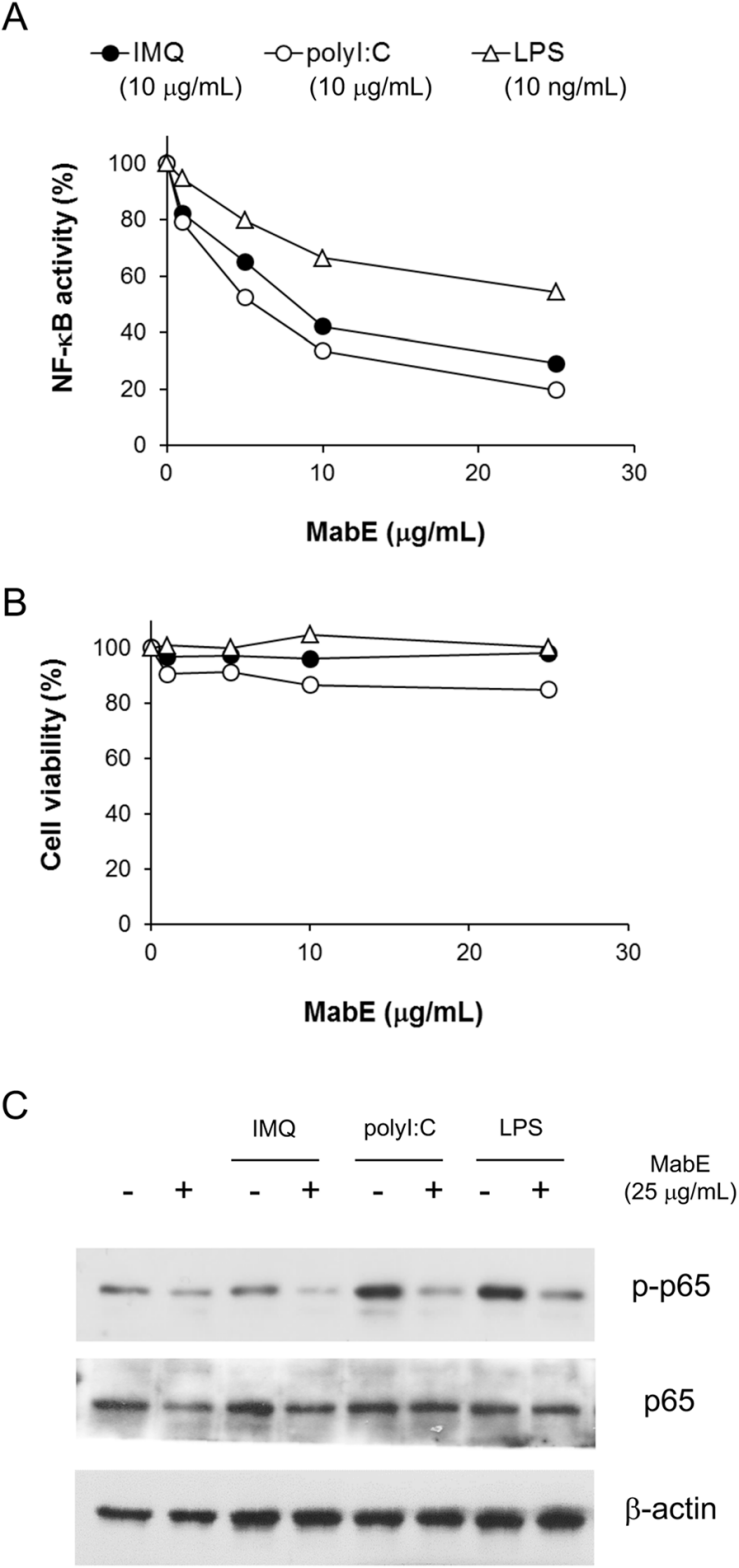
MabE inhibits the TLR ligand-induced activation of NF-κB in RAW264.7 cells. **a**, **b** RAW264.7-NFκB-Luc2 cells (5 × 10^4^ cells/well) were seeded onto 96-well plates and pretreated with MabE (1,5,10 or 25 μg/mL). After 1 h, they were stimulated with each TLR ligand (IMQ: 10 μg/mL, polyI:C: 10 μg/mL or LPS: 10 ng/mL). Luciferase activity (6 h) or cell viability (24 h) was measured, and the relative activity or viability compared with untreated control cells was assessed. **c** RAW264.7 cells (1 × 10^6^ cells/well) were seeded onto 6-well plate and pretreated with MabE (25 μg/mL) or culture medium. After 1 h, they were stimulated with each TLR ligand (IMQ: 20 μg/mL, polyI:C: 20 μg/mL or LPS: 100 ng/mL) for 3 h. Equal amounts of protein in cell lysates were analyzed by Western blotting. The β-actin protein levels were used to confirm that equal amounts of protein were subjected to electrophoresis

To first investigate the effects of MabE on TLR ligand-induced NF-κB activation, we used the RAW264.7-NFκB-Luc2 cell line and several TLR ligands. As shown in Supplemental Figure 1, transcriptional activity of NF-κB increased after stimulation with different TLR ligands, polyI:C (TLR3 ligand), lipopolysaccharide (LPS, TLR4 ligand) and IMQ (TLR7 ligand) in a dose- and time-dependent manner. Using the dose and time of each TLR ligand yielding similar degrees of NF-κB activity, we examined the effects of MabE on TLR ligand stimulation in RAW264.7 cells. As shown in Fig. 1a, MabE treatment at the indicated dose suppressed all TLR ligand-induced luciferase reporter activity in RAW264.7-NFκB-Luc2 cells. Although MabE did not affect the viability of RAW264.7-NFκB-Luc2 cells under the same condition (Fig. 1b), the phosphorylation of the p65 subunit of NF-κB induced by TLR ligands was inhibited (Fig. 1c). In addition to the activation of the NF-κB pathway, the mitogen-activated protein kinase (MAPK) signaling pathway is widely recognized as another important downstream signaling pathway in TLR ligand-stimulation. As shown in Supplementary Figure 2, the phosphorylation of p38 and ERK induced by TLR ligands was also suppressed by MabE.

**Fig. 2:**
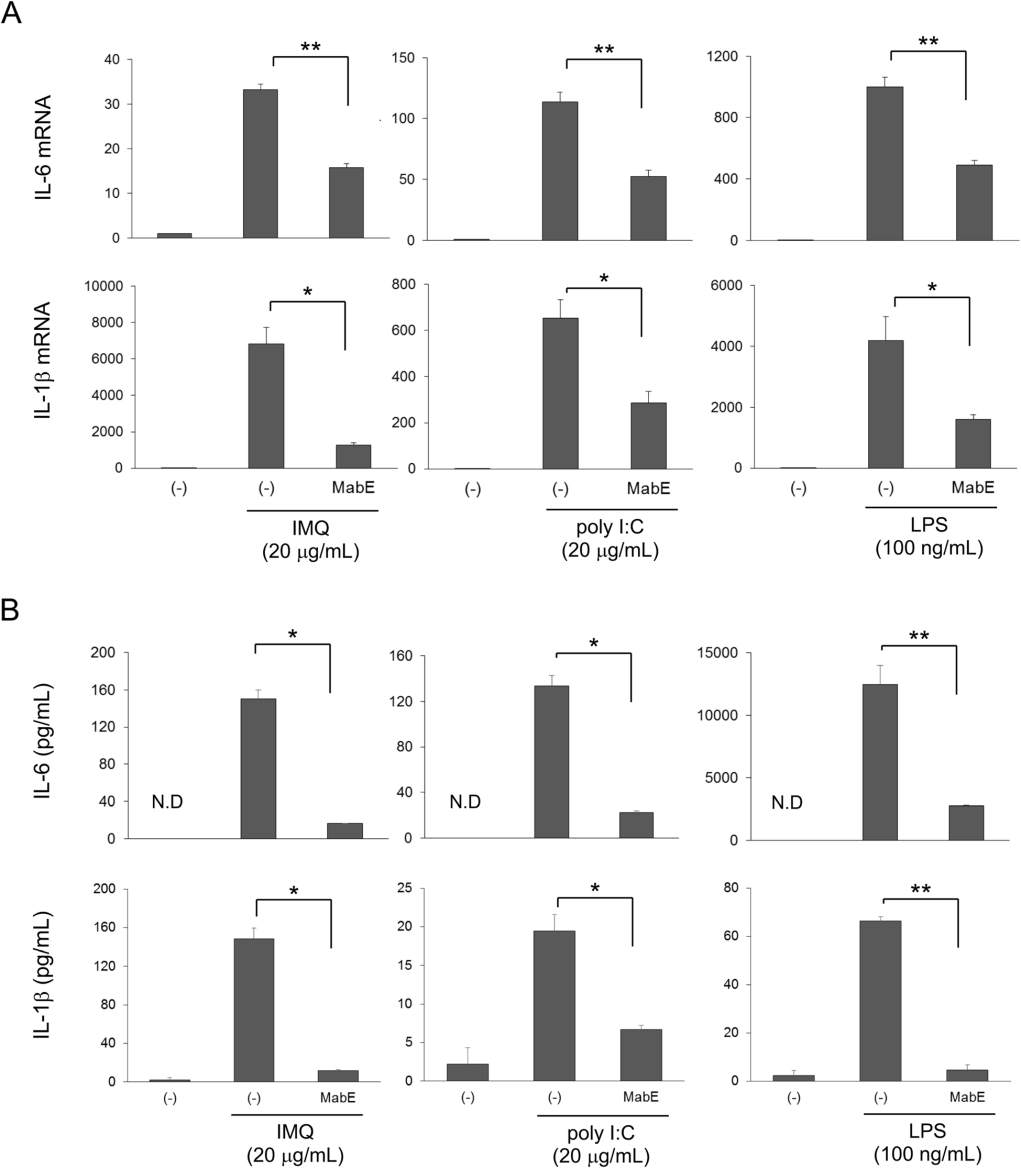
Effects of MabE on TLR ligand-induced inflammatory cytokine production in RAW264.7 cells. **a** RAW264.7 cells were treated with MabE (25 μg/mL) for 1 h and then stimulated with IMQ (20 μg/mL), poly I:C (20 μg/mL) or LPS (100 ng/mL) for 3 h. The mRNA expression levels of interleukin (IL)-6 and IL-1β were quantified by real-time PCR. **b** The production of IL-6 and IL-1β was measured in the culture medium of cells stimulated with IMQ (20 μg/mL), poly I:C (20 μg/mL) or LPS (100 ng/mL) for 24 h in the presence of 25 μg/mL of MabE. After culture supernatants were collected, each cytokine was quantified using the ELISA kit according to the manufacturer’s instructions. N.D: not detectable

Following the activation of TLRs, innate immune cells produce pro-inflammatory cytokines, such as IL-6 and IL-1, to initiate and maintain inflammation; therefore, we next examined the effects of MabE on the production of IL-6 and IL-1 from RAW264.7 cells induced by TLR ligands. Although the ligands for each TLR induced the mRNA expression (Fig. 2a) and protein production (Fig. 2b) of IL-6 and IL-1β, MabE significantly suppressed the production of IL-6 and IL-1β at both mRNA and protein levels (Fig. 2). Collectively, these results suggest that MabE suppresses the activation of both NF-κB and MAPK inflammatory pathways, and the production of IL-6 and IL-1β in RAW264.7 cells induced by TLR ligands.

### Moracin O and P inhibits the TLR ligand-induced NF-κB activation in RAW264.7 cells

**Fig. 3:**
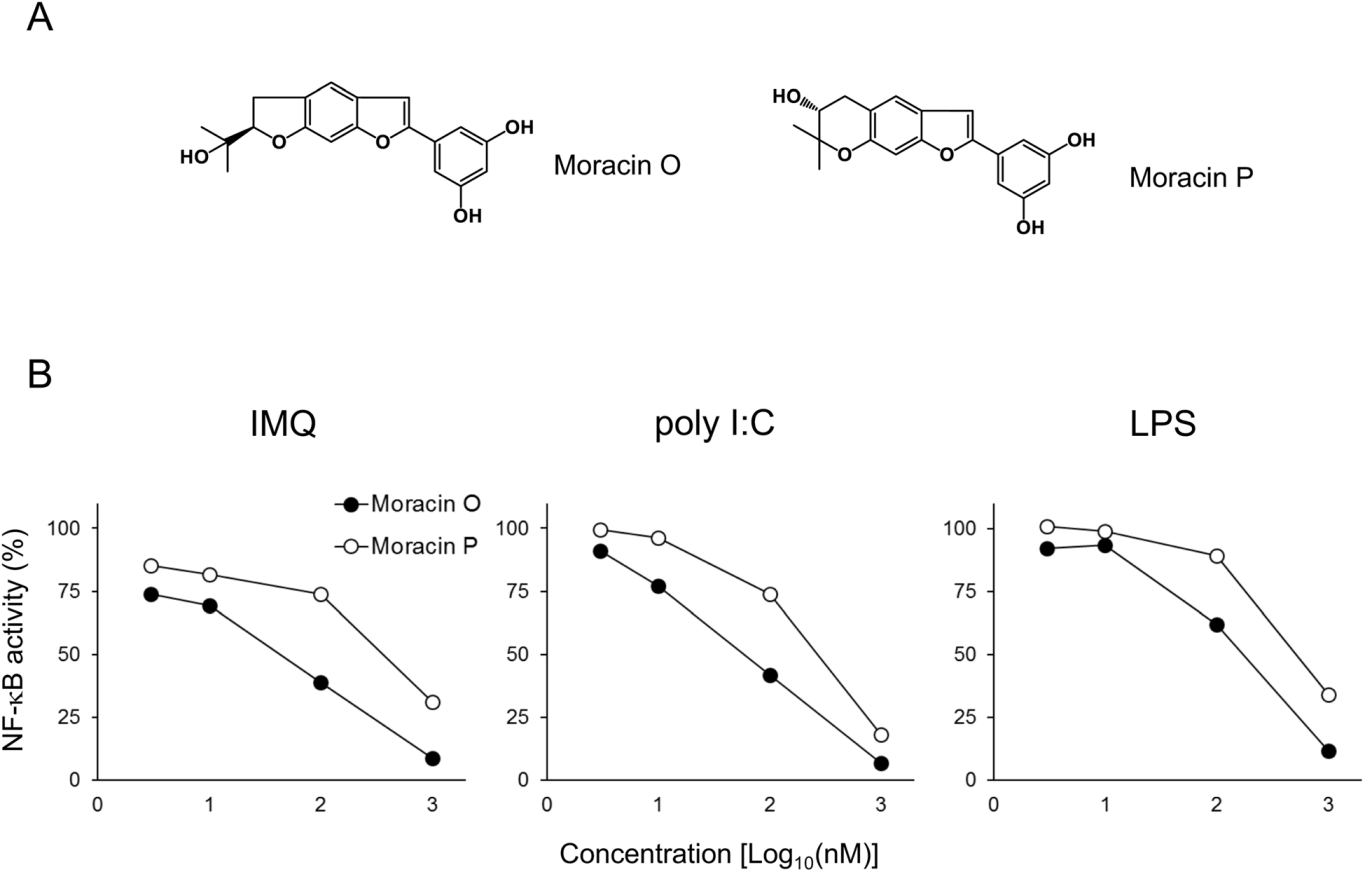
Moracin O and P inhibit the TLR ligand-induced NF-κB activation in RAW264.7 cells. **a** Chemical structure of Moracin O and Moracin P. **b** RAW264.7-NFκB-Luc2 cells (5 × 10^4^ cells/well) were seeded onto 96-well plates and pretreated with Moracin O or Moracin P (0.3,3,10,100 or1000 nM). After 1 h, they were stimulated with each TLR ligand (IMQ: 10 μg/mL, polyI:C: 10 μg/mL or LPS: 10 ng/mL) for 6 h. The activity of NF-κB was measured using IVIS imaging system. The inhibitory effects on NF-κB activation were assessed relative to NF-κB activation in untreated control

In a previous study, we identified Moracin O and P (Fig. 3a) as two major responsible compounds isolated from MabE to protect against keratinocyte damage induced by TRAIL [21]. To examine whether Moracin O and P are active constituents of MabE to inhibit TLR ligand-induced inflammatory signals, we assessed their effect in the luciferase reporter assay using RAW264.7-NFκB-Luc2 cells stimulated with TLR ligands. As shown in Fig. 3b, both Moracin O and P strongly inhibited the activation of NF-κB induced by different TLR ligands, polyI:C, LPS and IMQ, at a similar degree. Therefore, we conclude that Moracin O and P are also responsible for the inhibitory effects of MabE on TLR ligand-induced inflammatory signals.

### MabE inhibits IMQ-induced skin inflammation

**Fig. 4:**
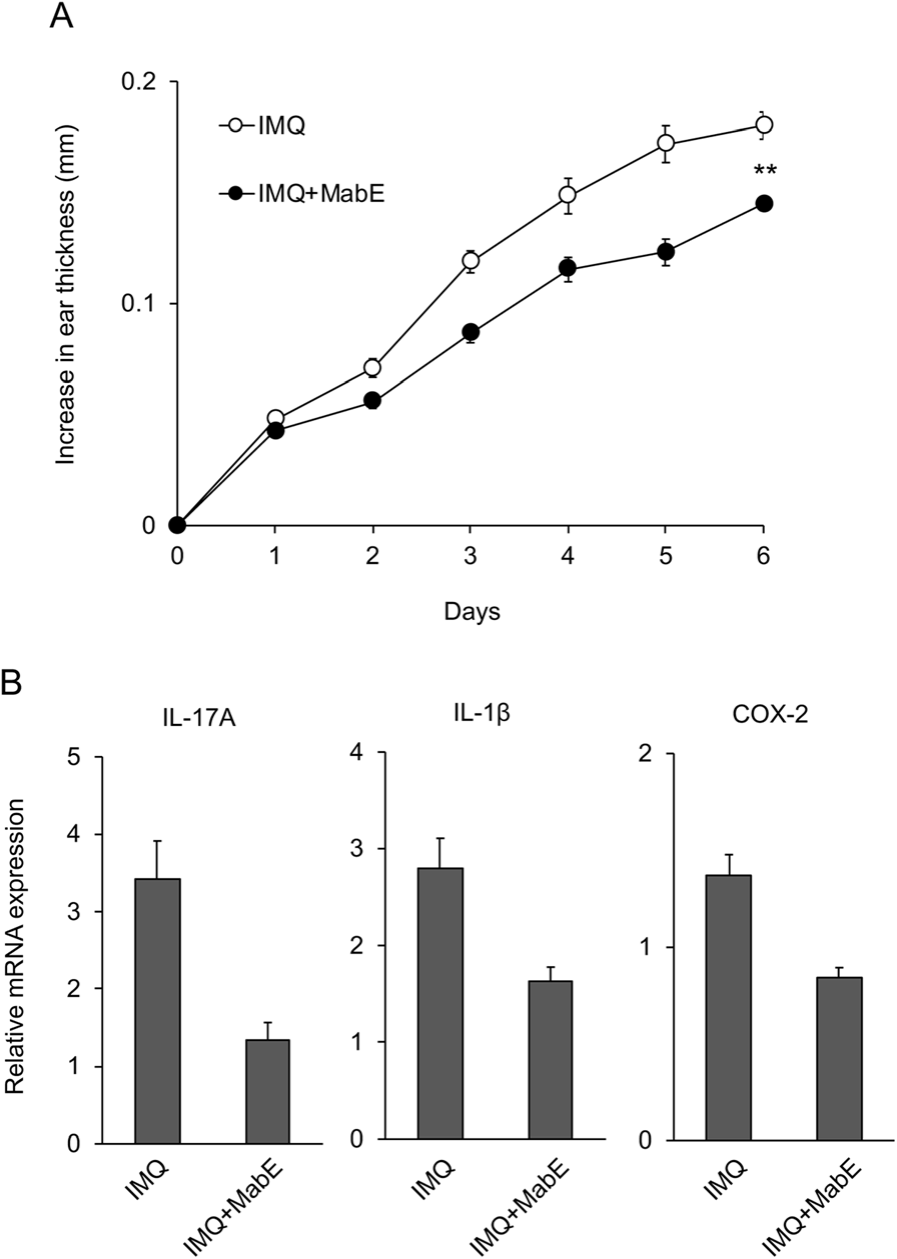
MabE inhibits IMQ-induced skin inflammation. Balb/c mice received a daily topical dose of IMQ cream (5%, 20 μg) on the right ear for 5 consecutive days (*n* = 6, each group). A group of mice was treated with MabE (200 μg in 20 μL of 50% EtOH) on the right ear 30 min before IMQ challenge. **a** The ear thickness of the right ear was measured on the days indicated. **b** On day 6, mice were sacrificed and skin biopsies were performed. Relative mRNA expression of the indicated genes is shown. The data indicate normalized mRNA expression at each time point compared with untreated control mice (day 0, *n* = 4 mice/ group). Data are presented as the mean ± SEM. *p < 0.05, **p < 0.01. The presented data are representative of three independent experiments

To further examine the anti-inflammatory effects of MabE under the pathological condition of TLR activation, we used an IMQ-induced skin inflammation model, which is known as a clinically relevant mouse model of psoriasis. To investigate the effect of MabE in this model, mice were topically treated with MabE on their ears before IMQ challenge. As shown in Fig. 4, MabE treatment inhibited the ear swelling of IMQ-treated mice, in addition to the mRNA expression of IL-17A, IL-1β and COX-2, which are known to be involved in the development and/or exacerbation of IMQ-induced psoriasis. This suggests that MabE inhibits psoriatic skin inflammation and the suppresses inflammatory gene expression.

### MabE inhibits IMQ-induced activation of IL-17A-producing γδT cells

**Fig. 5:**
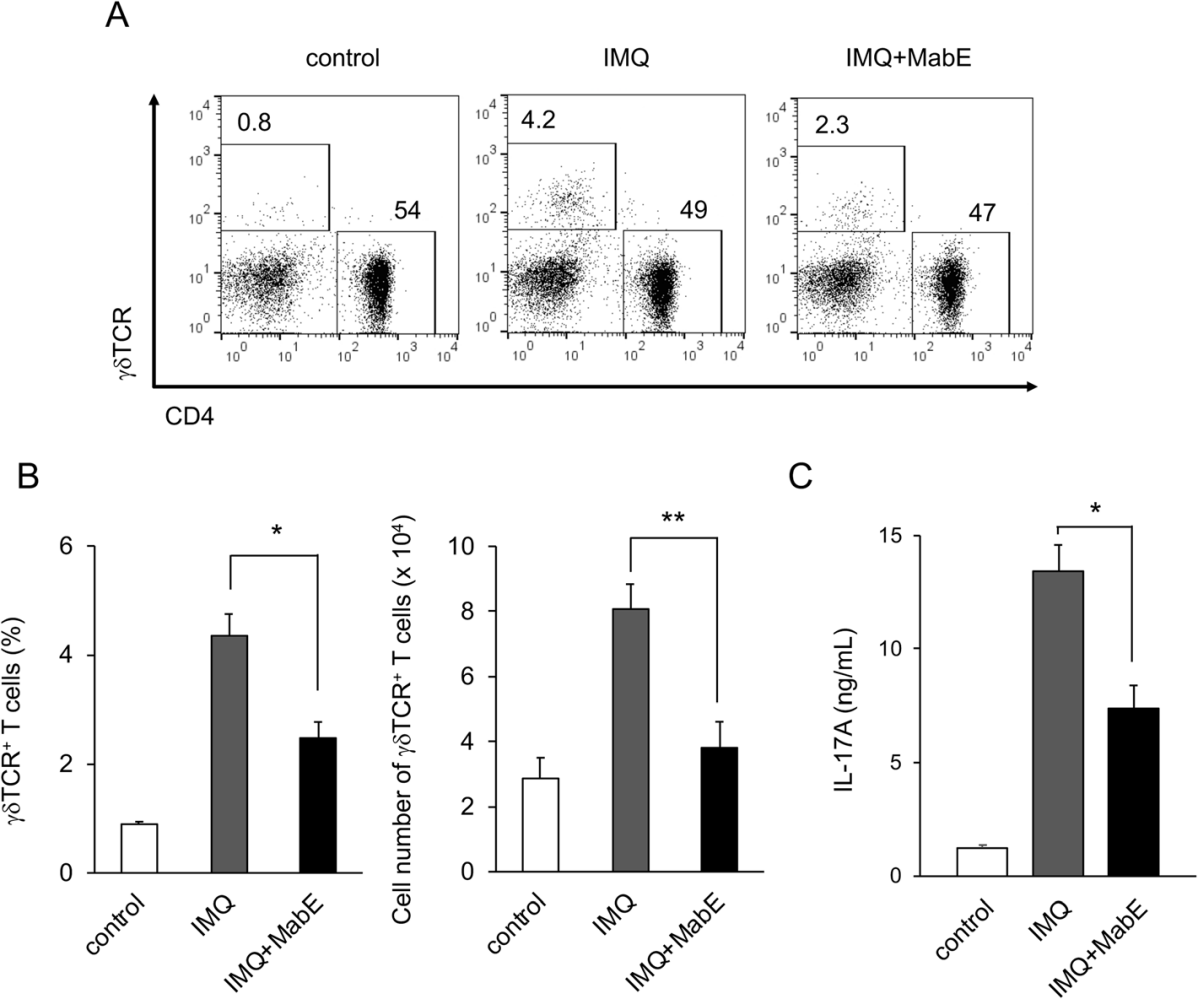
MabE inhibits IMQ-induced activation of IL-17A-producing γδT cells. Balb/c mice were treated with IMQ cream for 6 consecutive days and splenocytes were collected. **a** Cells were subjected to flow cytometry analysis and the representative dot plots of lymphocytes electronically gated on CD3ε^+^ cells are shown. The numbers are the percentages of cells electronically gated on γδT cells (γδTCR^+^ CD4^−^) and CD4^+^T cells (γδTCR ^−^ CD4^+^). The summary (*n* = 4 mice/ group) of percentages (**b**) and cell numbers (**c**) of γδT cells is shown. **d** Cells were (2 × 10^6^ cells/well) stimulated with PMA (10 ng/mL) and Ionomycin (500 ng/mL) for 24 h. The cell-free culture supernatants were collected and the production of IL-17A was measured by ELISA. Data are presented as the mean ± SEM. *p < 0.05, **p < 0.01 compared with IMQ-treated mice. The presented data are representative of three independent experiments

As IL-17A produced by γδT cells is essential for the development of IMQ-induced psoriasis, we next investigated the effects of MabE on the IL-17A-producing γδT cells. IMQ-challenged mice were treated with or without MabE for 6 days, and splenocytes were collected for flow cytometry analysis. As shown in Fig. 5a and b, IMQ-treated mice exhibited a significant increase in splenic γδT cells compared with untreated control mice. Of note, MabE treatment inhibited this increase in γδT cells after IMQ treatment (Fig. 5a and b). Although the production of IL-17A from the splenocytes was significantly induced by IMQ-challenge, it was significantly inhibited by MabE. In addition to the systemic inhibition of IL-17A production from splenocytes of IMQ-treated mice, MabE also directly inhibited the production of IL-17A from the splenocytes stimulated with PMA and ionomycin A in a dose-dependent manner (Supplementary Fig. 3). Collectively, these results suggest that the topical treatment of MabE inhibited IMQ-induced skin inflammation, possibly through the systemic activation of IL-17A-producing γδT cells.

## Discussion

Antigen-presenting cells, such as macrophages or dendritic cells express numerous receptors on their surface, and play an important role in inducing immune responses in the early stage of infection [24]. Among these receptors, TLRs are widely expressed in many species, and are important molecules for innate and adaptive immune responses. In our previous study, we demonstrated the cytoprotective effects of MabE by suppressing TRAIL-induced cellular damage and NF-κB activity in a human keratinocyte cell line [21]; however, the anti-inflammatory effects of MabE remained unclear. In this study, we found that MabE suppresses the activation of TLRs in the RAW264.7 macrophage cell line and imiquimod-induced ear edema.

MabE exerted anti-inflammatory effects on TLR activation induced by three different ligands, polyI:C, LPS and IMQ, which are the ligands of TLR3, TLR4 and TLR7 respectively. TLR3 and TLR7 are expressed on the endosomal membrane, and TLR4 is expressed on the plasma membrane [25]. These three TLRs require different adaptor molecules for their activation. TLR3 recognizes dsRNA and uses a TRIF-dependent pathway, whereas TLR7 recognizes ssRNA and uses a MyD88-dependent pathway. TLR4 recognizes LPS and uses both pathways [13, 26, 27]. Considering that MabE inhibits the activation of inflammatory signals induced by different TLR ligands, its anti-inflammatory effects may not be dependent on the specific adaptor molecule or the location of TLR expression.

Although the NF-κB signaling pathway is known as a key molecular pathway for TLR ligand-induced inflammation, the activation of TLRs also activates the MAPK signaling pathway, which is involved in many cellular functions [28–30]. Our present study demonstrated that MabE inhibits the phosphorylation of p38 and ERK1/2 in addition to phosphorylation of p65; therefore, MabE can inhibit both TLR ligand-induced activation of MAPK and NF-κB signaling pathways. Moreover, as Moracin O and P also strongly suppressed the activation of NF-κB induced by TLR ligands in RAW246.7 cells, these two compounds may be major active components of MabE to exert its anti-inflammatory effects.

IMQ-induced skin inflammation shares common characteristics with human psoriasis, including thes hyperproliferation of keratinocytes, erythema, scaling, acanthosis and infiltration of immune cells. It was previously reported that the inflammatory cytokine IL-17A is important in both human psoriasis and IMQ-induced skin inflammation [31], and the ear edema induced by topical application of IMQ was significantly attenuated in IL-17A knockout mice (data not shown). Although different types of immune cells, such as CD4^+^, CD8^+^, γδT, NKT cells and innate lymphocyte cells 3 (ILC3) are known to produce IL-17A [32], there is evidences to support that an IL-17-producing subset of CD4^+^ T (Th17) cells or γδT (γδT17) cells is a major source of IL-17A and essential for the development of inflammatory diseases, including IMQ-induced psoriasis [23, 33, 34]. Consistent with the suppression of ear edema induced by IMQ, MabE suppressed the systemic increase in γδT cells and IL-17A production in IMQ-treated mice. As the production of IL-17A production from naïve splenocytes stimulated with PMA/ionomycin was also suppressed by MabE (Supplementary Figure 3) and there was no change in CD4^+^ T cells in MabE-treated mice (data not shown), MabE may exert its anti-inflammatory effects in IMQ-treated mice partly through the suppression of γδT17 cell activation and their IL-17A production to attenuate psoriasis-like inflammation.

## Conclusion

Our study demonstrated the anti-inflammatory effects of MabE on the activation of the macrophage cell line RAW246.7 by TLRs and IMQ-induced psoriasis potentially through the inhibition γδT17 cells. In addition to Moracin O and P, which are the active constituents of MabE, there are many other phytochemicals isolated from *Morus alba* L. bark that exhibit anti-inflammatory effect; therefore, the combination of these compounds may be account for the potent pharmacological effects of *Morus alba* L. bark. As MabE also protected keratinocytes form cell death induced by inflammatory stimulation, its clinical use for skin protection in inflammatory dermatitis, such as atopic eczema or psoriasis, is expected.

## Supplementary Information

**Additional file 1: Supplementary Figure 1.** Response of RAW264.7-NFkB-Luc2 cells to different TLR ligands. TLR stimulation-induced activation of the NF-kB pathway in RAW264.7-NFkB-Luc2 cells. Cells (5 × 10^4^ cells/well) were seeded onto 96-well plates and stimulated with IMQ, polyI:C or LPS for 0–6 h. Luciferase activity was measured at the indicated time and the relative activity compared with untreated control cells was measured. **Supplementary Figure 2.** MabE inhibits the TLR ligand-induced activation of MAPK signals in RAW264.7 cells. RAW264.7 cells (10^6^ cells/well) were seeded onto 6-well plates and pretreated with MabE (25 mg/mL) or culture medium. After 1 h, they were stimulated with each TLR ligands (IMQ: 20 mg/mL, polyI:C: 20 mg/mL or LPS: 100 ng/mL) for 3 h. Equal amounts of protein in cell lysates were analyzed by Western blotting. The b-actin protein levels were used to confirm that equal amounts of protein were subjected to electrophoresis. **Supplementary Figure 3.** MabE directly suppresses IL-17A production from splenocytes stimulated with PMA/ionomycin Naïve Balb/c splenocytes were incubated with or without MabE (50 or 100 mg/mL) for 1 h, and then stimulated with PMA (10 ng/mL) and ionomycin (500 ng/mL) for 24 h. The cell-free culture supernatants were collected and the IL-17A concentration was measured by ELISA. Data are presented as the mean ± SEM. **p < 0.01. The presented data are representative of three independent experiments.

**Additional file 2.**

**Additional file 3.**

## Data Availability

The datasets used and analyzed during the current study are available from the corresponding author upon reasonable request.

## Abbreviations

NF-κB: Nuclear factor-kappa B
PRRs: pattern recognition receptors
TLRs: Toll-like receptors
LPS: Lipopolysaccharide
dsRNA: Double stranded RNA
ssRNA: single stranded RNA
DAMPs: Damage-associated molecular patterns
MAPK: Mitogen-activated protein kinase
IMQ: Imiquimod
TRIF: TIR-domain-containing adapter-inducing interferon-β
MyD88: Myeloid differentiation primary response 88
