# Supplementary material for: Anti-inflammatory effects of Morus alba Linne bark on the activation of toll-like receptors and imiquimod-induced ear edema in mice
Source: BMC Complement Med Ther. 2021 Apr 9;21:115. doi: 10.1186/s12906-021-03291-5 (PMC8033707; doi:10.1186/s12906-021-03291-5)
Supplement: Supplementary file 1 — Additional file 1: Supplementary Figure 1. Response of RAW264.7-NFkB-Luc2 cells to different TLR ligands. TLR stimulation-induced activation of the NF-kB pathway in RAW264.7-NFkB-Luc2 cells. Cells (5 × 10^4^ cells/well) were seeded onto 96-well plates and stimulated with IMQ, polyI:C or LPS for 0–6 h. Luciferase activity was measured at the indicated time and the relative activity compared with untreated control cells was measured. Supplementary Figure 2. MabE inhibits the TLR ligand-induced activation of MAPK signals in RAW264.7 cells. RAW264.7 cells (106 cells/well) were seeded onto 6-well plates and pretreated with MabE (25 mg/mL) or culture medium. After 1 h, they were stimulated with each TLR ligands (IMQ: 20 mg/mL, polyI:C: 20 mg/mL or LPS: 100 ng/mL) for 3 h. Equal amounts of protein in cell lysates were analyzed by Western blotting. The b-actin protein levels were used to confirm that equal amounts of protein were subjected to electrophoresis. Supplementary Figure 3. MabE directly suppresses IL-17A production from splenocytes stimulated with PMA/ionomycin Naïve Balb/c splenocytes were incubated with or without MabE (50 or 100 mg/mL) for 1 h, and then stimulated with PMA (10 ng/mL) and ionomycin (500 ng/mL) for 24 h. The cell-free culture supernatants were collected and the IL-17A concentration was measured by ELISA. Data are presented as the mean ± SEM. **p < 0.01. The presented data are representative of three independent experiments. [file 12906_2021_3291_MOESM1_ESM.pdf]

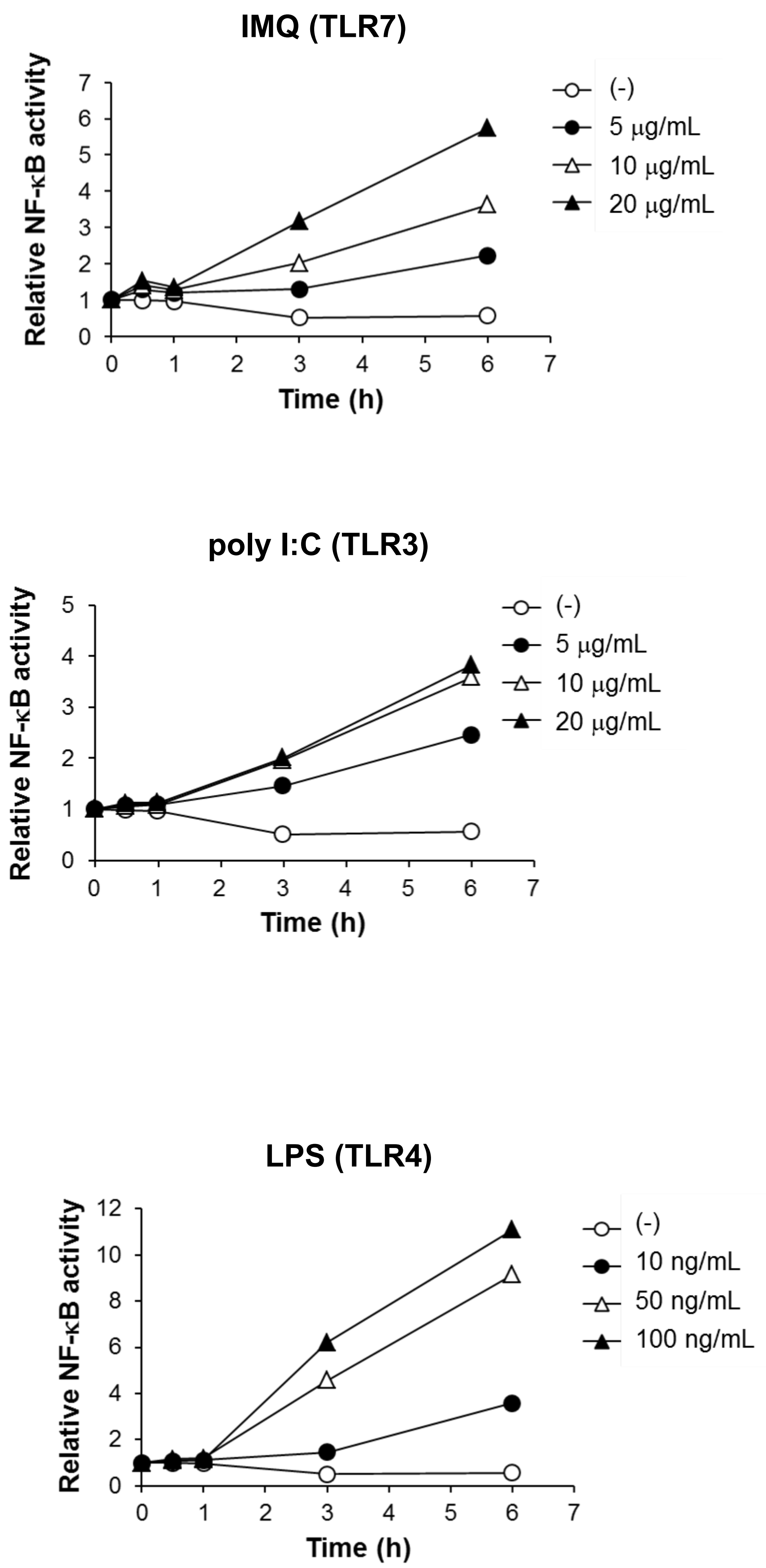

Supplementary Figure 1.

Response of RAW264.7-NFkB-Luc2 cells to different TLR ligands.

TLR stimulation-induced activation of the NF-kB pathway in RAW264.7-NFkB-Luc2 cells. Cells ($5x10^4$ cells/well) were seeded onto 96-well plates and stimulated with IMQ, polyI:C or LPS for 0-6 hours. Luciferase activity was measured at the indicated time and the relative activity compared with untreated control cells was measured.

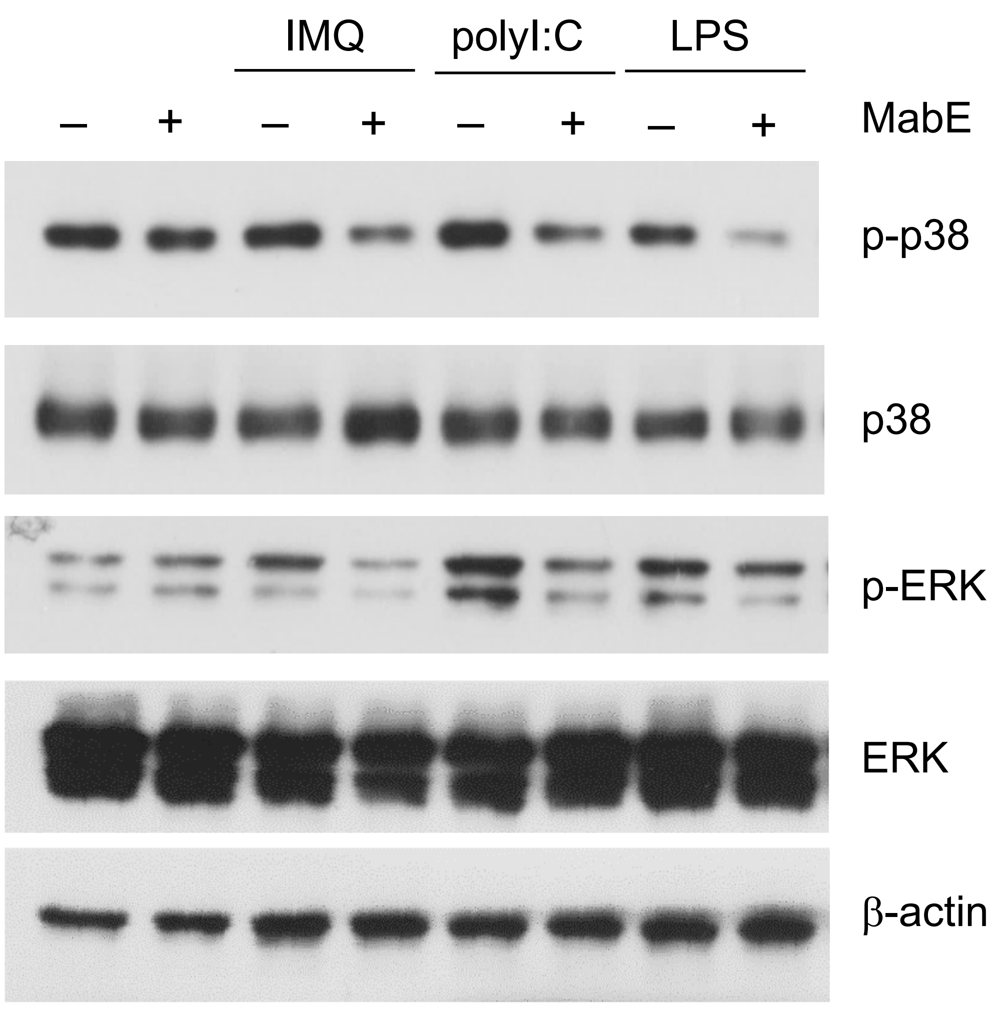

Supplementary Figure 2.

MabE inhibits the TLR ligand-induced activation of MAPK signals in RAW264.7 cells. RAW264.7 cells (10^6 cells/well) were seeded onto 6-well plates and pretreated with MabE (25 mg/mL) or culture medium. After 1 hour, they were stimulated with each TLR ligands (IMQ: 20 mg/mL, polyI:C: 20 mg/mL or LPS: 100 ng/mL) for 3 hours. Equal amounts of protein in cell lysates were analyzed by Western blotting. The b-actin protein levels were used to confirm that equal amounts of protein were subjected to electrophoresis.

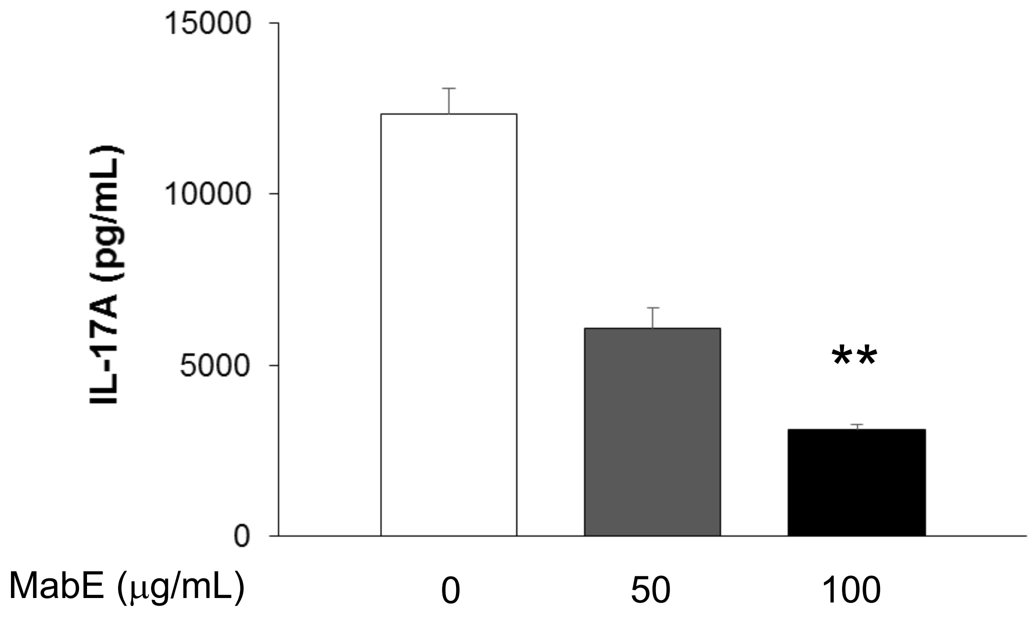

Supplementary Figure 3.

MabE directly suppresses IL-17A production from splenocytes stimulated with PMA/ionomycin

Naïve Balb/c splenocytes were incubated with or without MabE (50 or 100 mg/mL) for 1 hour, and then stimulated with PMA (10 ng/mL) and ionomycin (500 ng/mL) for 24 hours. The cell-free culture supernatants were collected and the IL-17A concentration was measured by ELISA. Data are presented as the mean ± SEM. **p<0.01. The presented data are representative of three independent experiments.
