# Supplementary material for: Anti-inflammatory effects of Morus alba Linne bark on the activation of toll-like receptors and imiquimod-induced ear edema in mice
Source: BMC Complement Med Ther. 2021 Apr 9;21:115. doi: 10.1186/s12906-021-03291-5 (PMC8033707; doi:10.1186/s12906-021-03291-5)
Supplement: Supplementary file 2 — Additional file 2. [file 12906_2021_3291_MOESM2_ESM.pptx]

## Slide 1
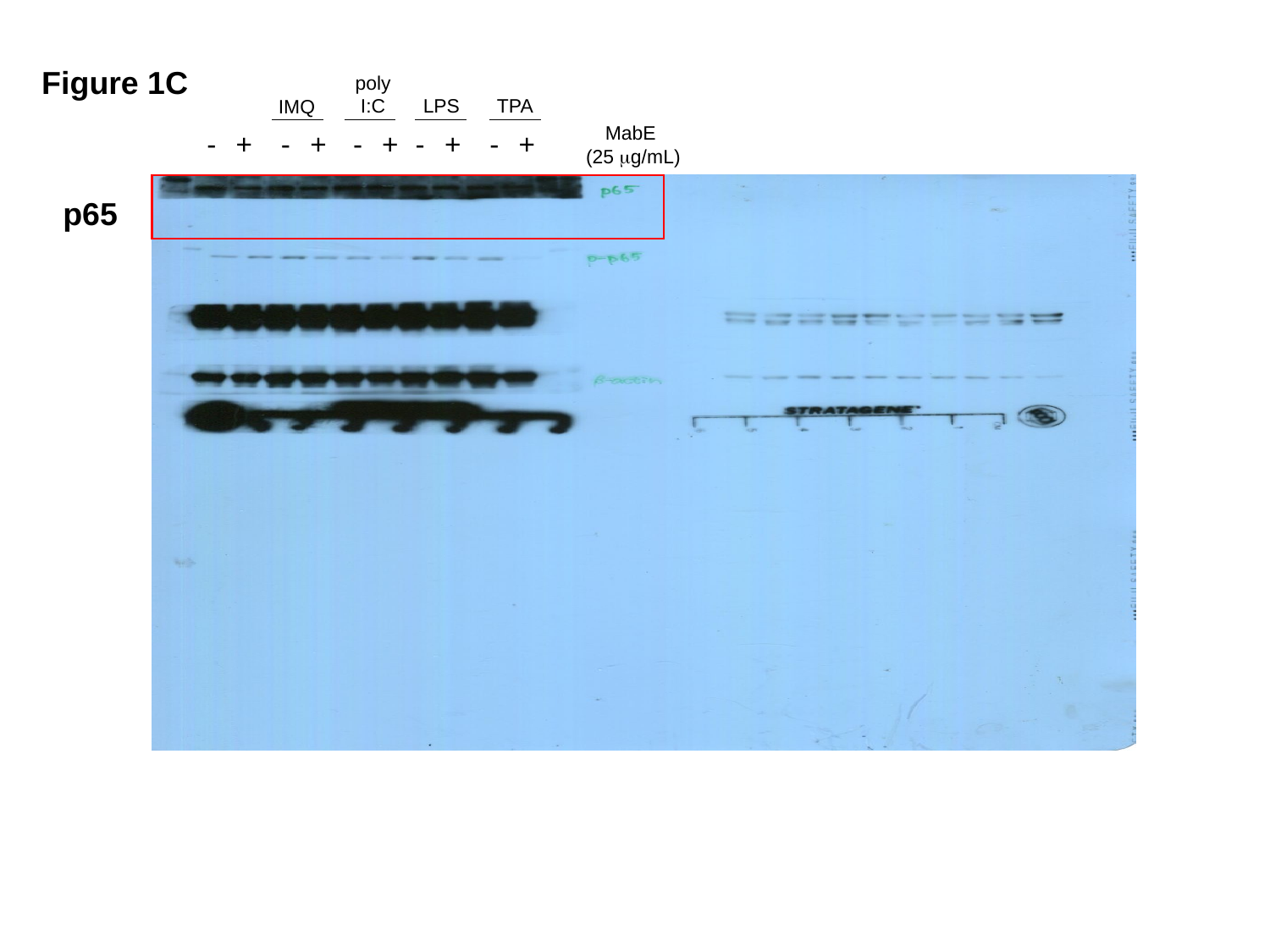

Figure 1C
poly I:C
LPS
TPA
IMQ
MabE
(25 mg/mL)
-
+
-
+
-
+
-
+
-
+
p65

## Slide 2
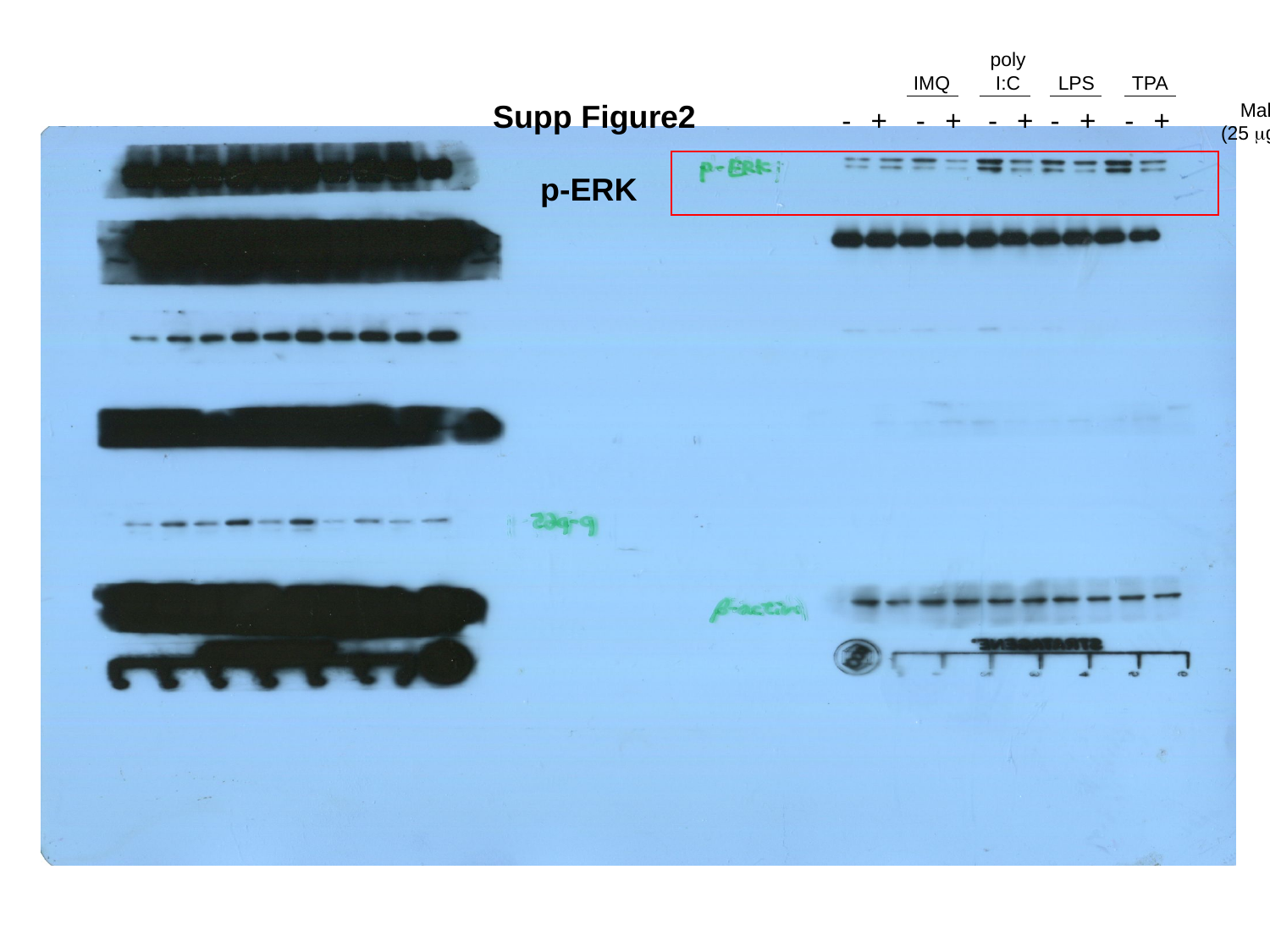

poly I:C
LPS
TPA
IMQ
MabE
(25 mg/mL)
-
+
-
+
-
+
-
+
-
+
Supp Figure2
p-ERK

## Slide 3
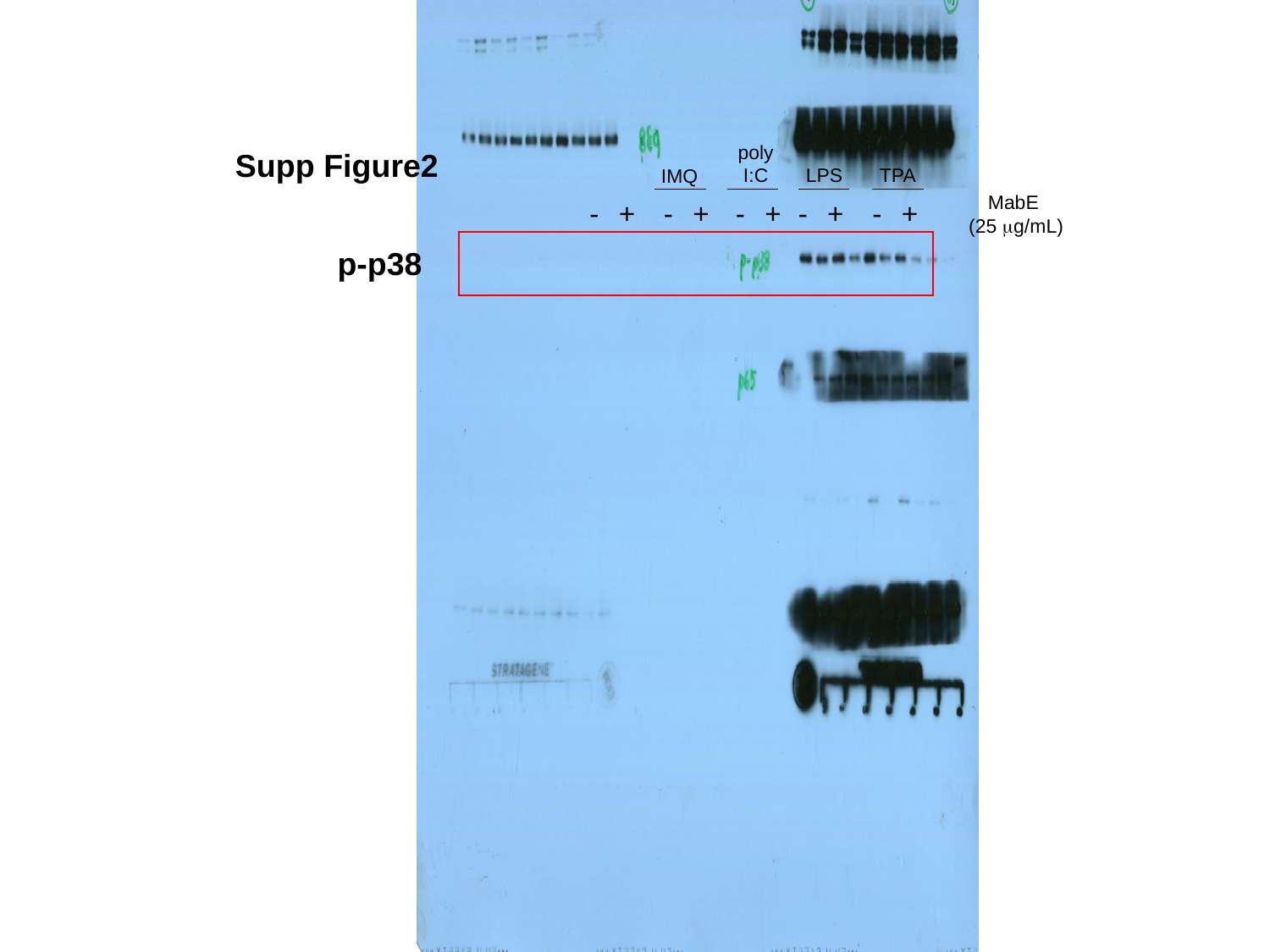

poly I:C
LPS
TPA
IMQ
MabE
(25 mg/mL)
-
+
-
+
-
+
-
+
-
+
Supp Figure2
p-p38

## Slide 4
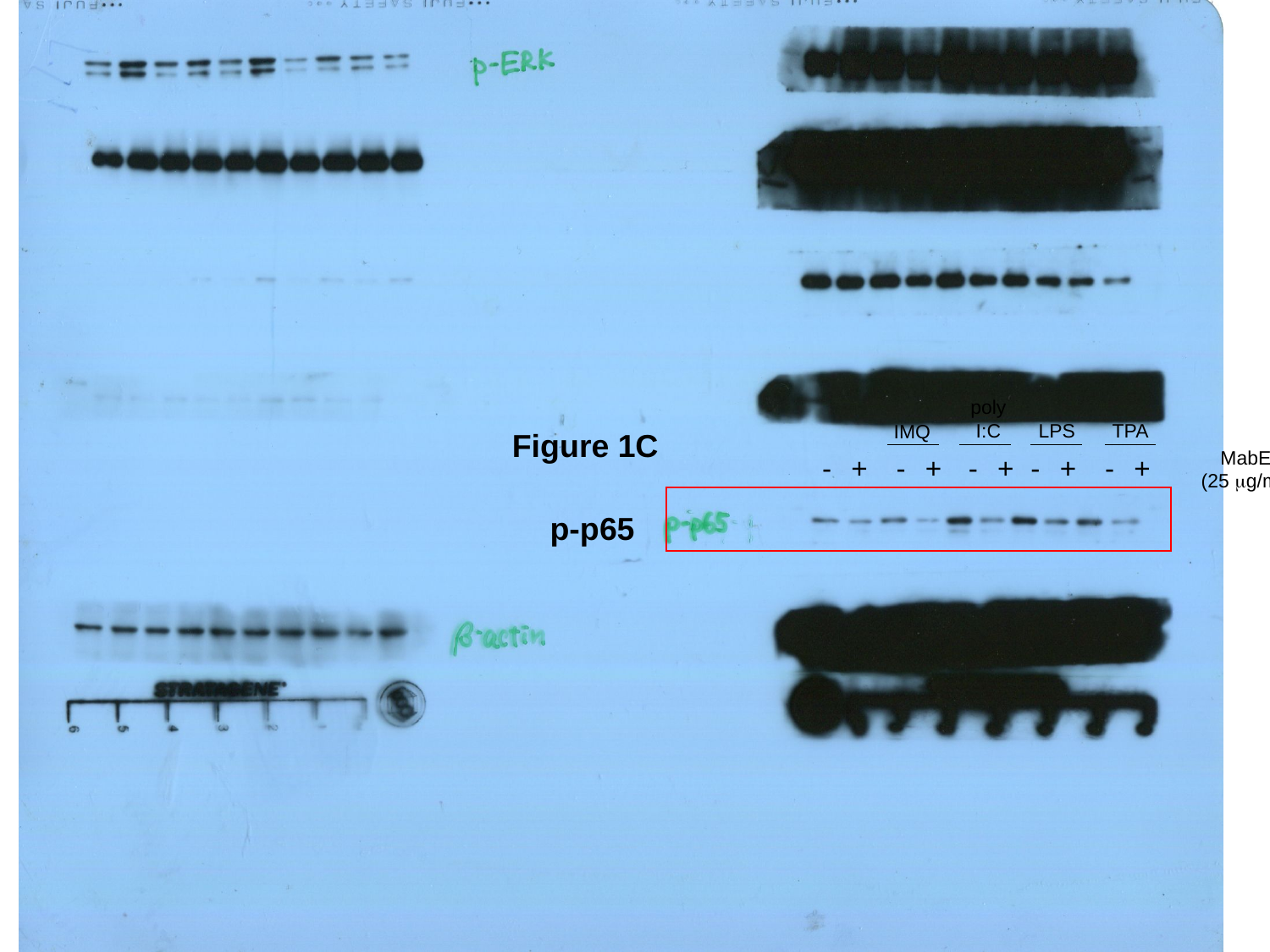

poly I:C
LPS
TPA
IMQ
MabE
(25 mg/mL)
-
+
-
+
-
+
-
+
-
+
Figure 1C
p-p65

## Slide 5
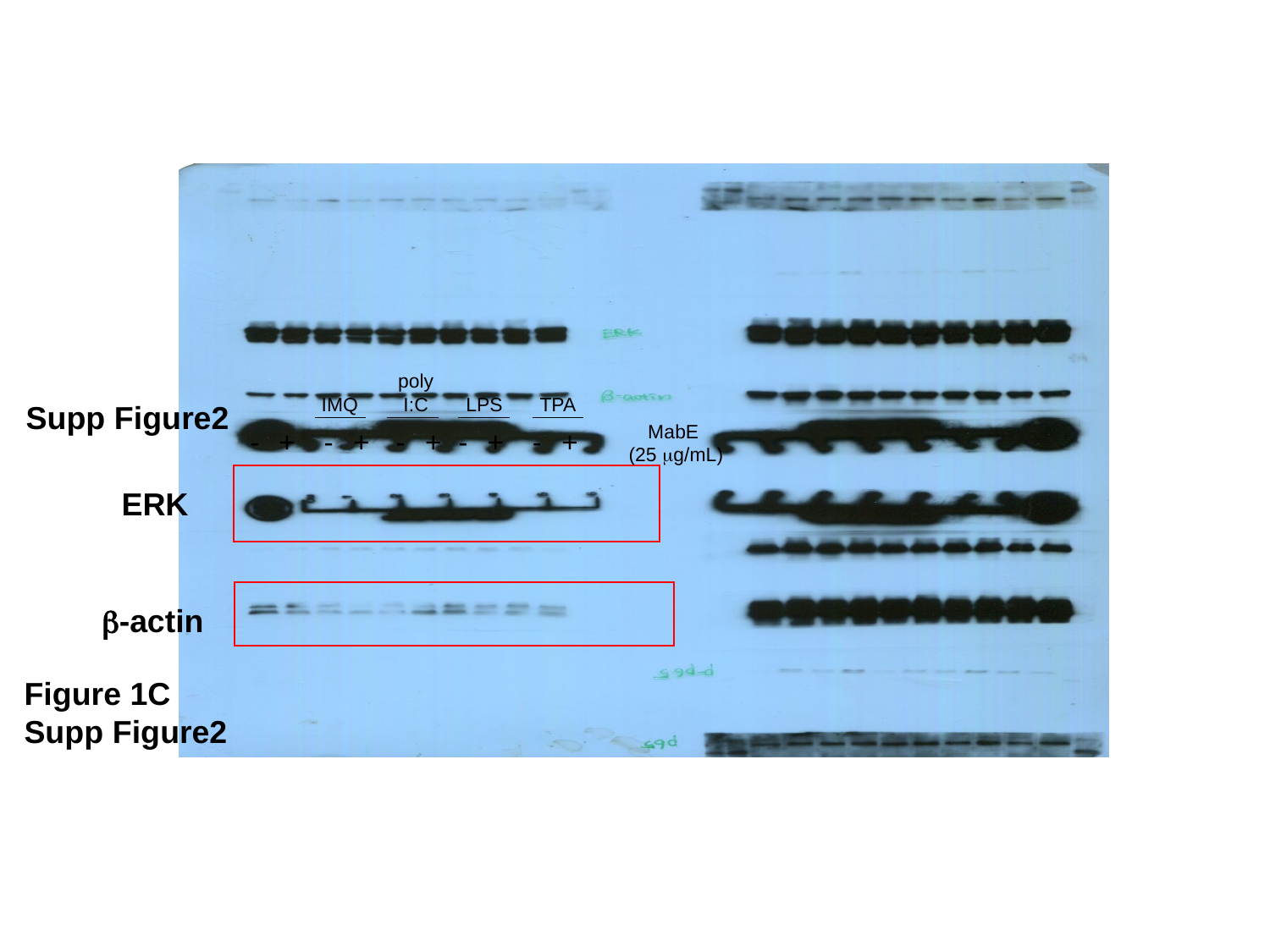

poly I:C
LPS
TPA
IMQ
MabE
(25 mg/mL)
-
+
-
+
-
+
-
+
-
+
Supp Figure2
ERK
b-actin
Figure 1C
Supp Figure2
