# Supplementary figures and images for: Anti-inflammatory effects of Morus alba Linne bark on the activation of toll-like receptors and imiquimod-induced ear edema in mice
Source: BMC Complement Med Ther. 2021 Apr 9;21:115. doi: 10.1186/s12906-021-03291-5 (PMC8033707; doi:10.1186/s12906-021-03291-5)

$\gamma\delta$ TCR

Control

IMQ

IMQ+MabE

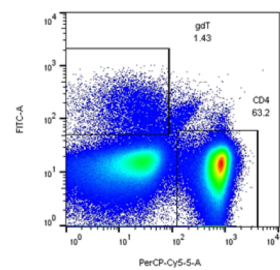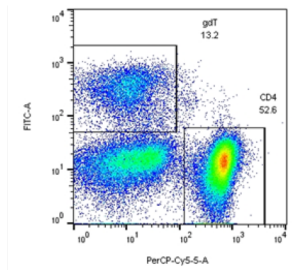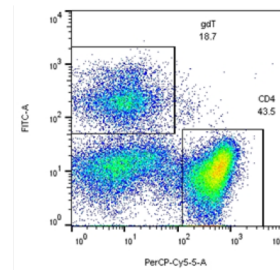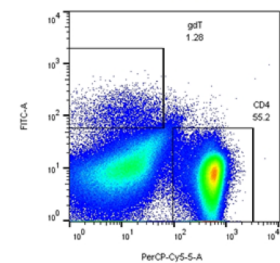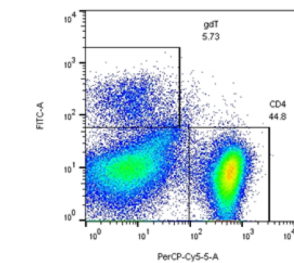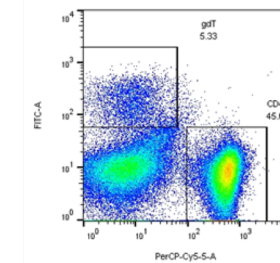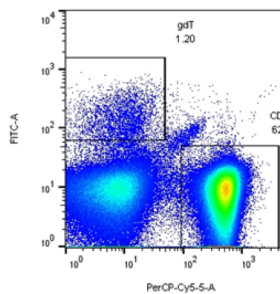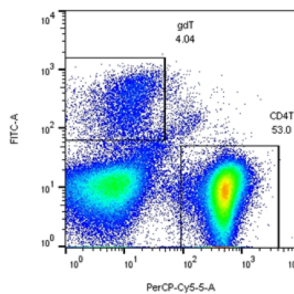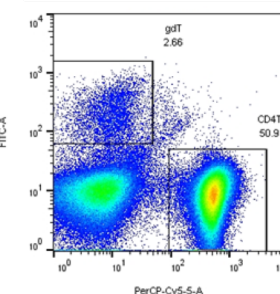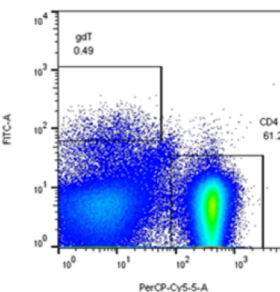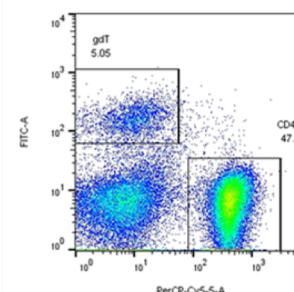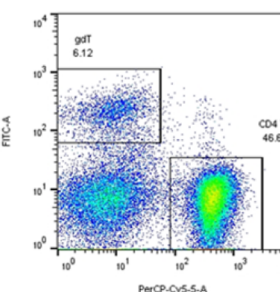

CD3

Supplement: Supplementary file 3 — Additional file 3. [file 12906_2021_3291_MOESM3_ESM.pdf]
